# Treatment of COVID-19 in a Patient With Maple Syrup Urine Disease

**DOI:** 10.7759/cureus.24368

**Published:** 2022-04-22

**Authors:** Kara F Morton, Ryan L Goetz, Kristin B Linscott, Nicholas J Van Wagoner

**Affiliations:** 1 Department of Medicine, University of Alabama at Birmingham, Birmingham, USA; 2 Department of Genetics, University of Alabama at Birmingham, Birmingham, USA

**Keywords:** amino acid, metabolic crisis, inherited metabolic disease, maple syrup urine disease, covid-19

## Abstract

Maple syrup urine disease (MSUD) is an inborn error of metabolism caused by a defect in the branched-chain alpha-ketoacid dehydrogenase complex (BCKDC). This leads to the accumulation of the branched-chain amino acids (BCAAs) leucine, isoleucine, and valine, which can cause neurotoxicity. Patients with MSUD are carefully managed from birth with dietary restrictions and can acutely decompensate in the setting of infections or injury.

We present the case of a 29-year-old female with a history of MSUD and rheumatoid arthritis on methotrexate and adalimumab who presented to our emergency department with symptoms suggestive of a metabolic crisis including nausea, vomiting, and presyncope. She was diagnosed with coronavirus disease 2019 (COVID-19) and admitted. An initial leucine level was mildly elevated at 253 μmol/L, consistent with her underlying metabolic condition. She was placed on an infusion of normal saline and 10% Dextrose (D10) in addition to a protein-restricted sick-day diet. Remdesivir therapy was initiated due to her immunocompromised status and high risk for decompensation but had to be discontinued due to nausea and vomiting that negatively impacted the patient’s oral intake. Her leucine level peaked at 647 μmol/L; however, her neurologic examination remained benign without signs of cerebral edema. With prompt involvement of our metabolic genetics team and initiation of intravenous fluids and the sick-day diet protocol, we avoided a metabolic crisis. The patient was discharged on day 5 of hospitalization with no complications from COVID-19 infection.

This case highlights the individualized approach to the treatment of COVID-19 infection in a patient with a metabolic disorder. COVID-19 infection in the setting of MSUD has only been reported in two prior publications, one being a severe metabolic crisis with neurologic involvement. Fortunately, our patient experienced a mild case of COVID-19 without significant respiratory symptoms, and we were able to prevent a metabolic crisis during admission.

## Introduction

Maple syrup urine disease (MSUD) is an autosomal recessive inborn error of metabolism characterized by a defect in the branched-chain alpha-ketoacid dehydrogenase complex (BCKDC) that affects about one in every 185,000 live births worldwide [1,2]. This enzymatic defect causes decreased functioning of the enzyme complex, leading to the buildup of the branched-chain amino acids (BCAAs) leucine, isoleucine, and valine as well as their corresponding alpha-ketoacids. When left untreated, an infant with MSUD will develop irritability, anorexia, dystonia, lethargy, and eventually coma and death secondary to brain edema from this buildup of metabolic compounds. If identified and treated early with a BCAA-restricted diet with the goal of preventing muscle protein catabolism, patients with MSUD typically live into adulthood [1]. Despite adequate dietary modification, a variety of insults including infection, surgery, trauma, and fasting can trigger a metabolic crisis in children and adults. A metabolic crisis in these individuals presents as encephalopathy, gastrointestinal disturbances, and lethargy [1,2]. The coronavirus disease 2019 (COVID-19) pandemic, which has infected nearly 80 million American citizens and killed over 900,000 according to data from the Centers for Disease Control (CDC), poses a serious threat to patients with MSUD who are prone to metabolic derangements [3]. We present the case of a young adult female who developed a mild COVID-19 infection and who experienced a favorable outcome.

## Case presentation

A 29-year-old female with MSUD and rheumatoid arthritis on methotrexate and adalimumab presented to the emergency department with one day of acute onset headache, dizziness, nausea, and non-bloody, non-bilious vomiting. This presentation was consistent with MSUD-related metabolic crises she has had in the past. She had been restricting her dietary protein intake at home prior to presentation. She contacted the on-call geneticist when her symptoms failed to resolve and was instructed to present to the emergency department. Vital signs on presentation were as follows: temperature, 99.1°F; heart rate, 106 beats per minute; respiratory rate, 21 breaths per minute; blood pressure, 126/86; and oxygen saturation, 97% on room air. A physical examination was only notable for dysarthria and decreased strength and tone in the bilateral lower extremities, consistent with her neurologic baseline. The remainder of the neurologic examination was benign and nonsuggestive of cerebral edema, one of the serious complications of an MSUD metabolic crisis. Basic laboratory values were only notable for elevated alkaline phosphatase of 139 u/L (reference range: 37-117 u/L). A complete blood count was normal with a differential notable for lymphocytopenia. An amino acid panel was obtained (Table 1) and demonstrated a leucine level that was mildly elevated at 253 μmol/L, which did not support a metabolic crisis and was instead consistent with her underlying metabolic condition. A COVID-19 test was positive. A chest X-ray was clear. Although the patient’s initial amino acid panel was not suggestive of a metabolic crisis, given her ongoing nausea and vomiting with poor oral intake in the setting of COVID-19 infection, she was admitted to the general internal medicine floor. She was initiated on remdesivir per the Pinetree protocol [4] due to her immunocompromised state from biologic pharmacotherapy for the treatment of rheumatoid arthritis.

**Table 1:** Amino acid panel on the day of admission compared to day 2 of hospitalization. ND: not detected, H: high, L: low

Amino acid	Day of admission value (µmol/L)	Day 2 of hospitalization value (µmol/L)
Aspartic acid	9	19 (H)
Threonine	86	121
Serine	86	110
Asparagine	45	64
Glutamic acid	29	150 (H)
Glutamine	520	368 (L)
Alpha-aminoadipic acid	6	5
Glycine	213	266
Alanine	172 (L)	190 (L)
Citrulline	21	19
Aminobutyric	ND	4
Valine	187	507 (H)
Cysteine	31	29
Methionine	9	10
Cystathionine	ND	ND
Isoleucine	81	244 (H)
Leucine	253 (H)	647 (H)
Tyrosine	27	40
Phenylalanine	41	66
Homocysteine	ND	ND
Ornithine	63	94
Lysine	107	122
1-Methylhistidine	8	10
Histidine	39 (L)	66
3-Methylhistidine	3	ND
Arginine	21	9 (L)
Hydroxyproline	7	20 (H)
Proline	144	118

After admission, the metabolic genetics team was promptly involved for assistance with the management and prevention of a metabolic crisis. They initiated an infusion of normal saline with 10% Dextrose (D10) along with the sick-day diet protocol, which is a dietary intervention that aims to prevent skeletal muscle catabolism and promote protein anabolism. The sick-day diet prescription typically involves increasing daily caloric intake to 120%-150% of daily energy expenditure with a restriction of leucine intake by 50%-100% [1,5]. Our patient was given a protein-free diet alongside the normal saline D10 maintenance fluid infusion. Dextrose and isotonic crystalloid infusion were utilized with two aims: to prevent catabolism with high caloric intake and to prevent cerebral edema with the use of mildly hypertonic normal saline. While dextrose is appropriate to prevent catabolism early in admission, insufficient protein intake can also lead to catabolism in the long run, and so, the sick-day diet protocol aims to slowly reintroduce protein into the diet. Her neurologic examination remained at baseline, and she did not complain of respiratory symptoms. On days 2 and 3 of hospitalization, she continued to complain of diarrhea and persistent nausea with intermittent emesis despite the utilization of as-needed intravenous ondansetron. She was unable to consistently finish the sick-day meals provided to her. This decreased oral intake was concerning due to the risk of catabolism it posed. On day 2, a repeat amino acid panel was drawn (Table 1) and demonstrated an elevated leucine level of 647 μmol/L, an elevated valine level of 507 μmol/L, and an elevated isoleucine level of 244 μmol/L. A repeat neurologic examination demonstrated no significant changes from baseline, which suggested that the rate of leucine production was not overly rapid. However, the elevation in BCAA levels was concerning for impending metabolic crisis in the setting of continued emesis and poor oral intake. Nausea is one of the most common side effects of remdesivir [4], so the decision was made to discontinue the medication on day 3 of hospitalization.

The patient experienced resolution of nausea and vomiting and was able to tolerate improved oral intake. She was gradually weaned from D10 maintenance fluids as her oral intake and gastrointestinal symptoms improved on days 2-4 of hospitalization. Her daily protein intake was also gradually increased per sick-day diet protocol [5]. Her neurologic status remained at baseline with no evidence of cerebral edema throughout her hospital stay. She never developed respiratory symptoms as a result of the COVID-19 infection and remained stable on room air. On the fifth day of hospitalization, she was stable for discharge with close follow-up with her genetics team. She was instructed to continue to wear a mask and socially distance herself in public settings for at least five more days per current CDC guidelines. On follow-up with her metabolic geneticist five days post-discharge, she was doing very well and tolerating her usual oral intake.

## Discussion

**Figure 1:**
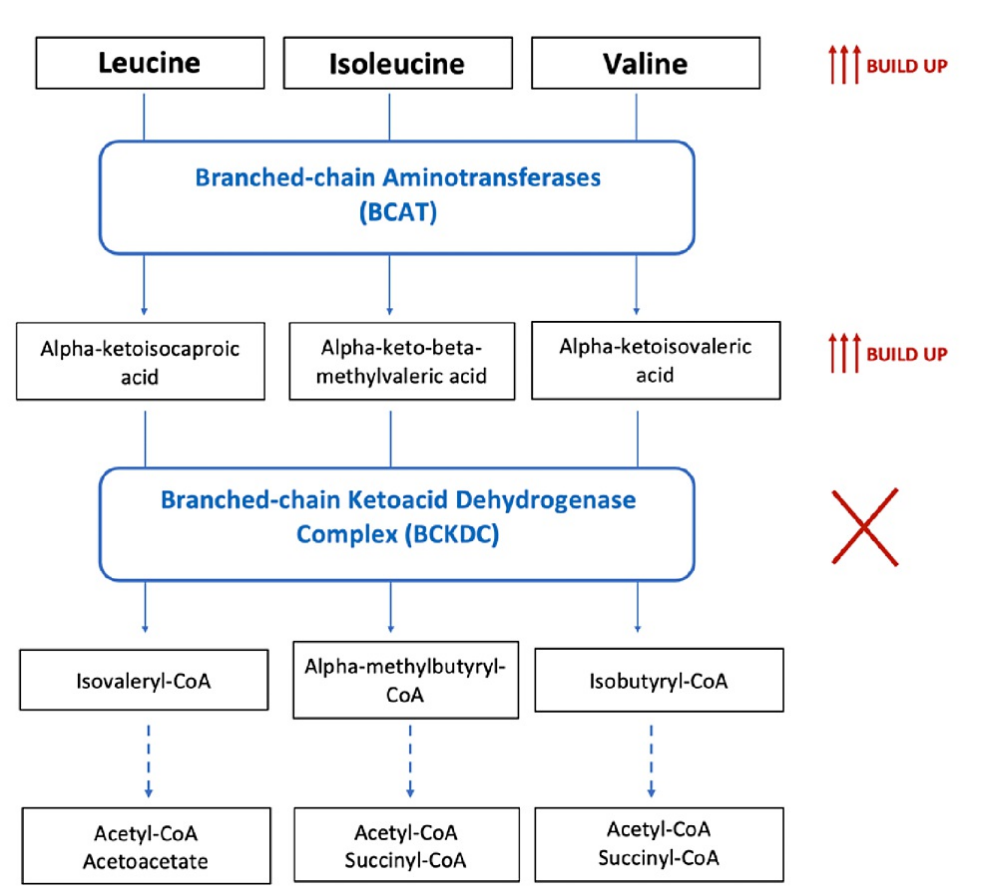
Overview of the effect of MSUD on the catabolic pathway for BCAAs leucine, isoleucine, and valine. After a transamination catalyzed by the branched-chain aminotransferase, three corresponding alpha-ketoacids are produced. In MSUD, the BCKDC is not effective, and these alpha-ketoacids cannot undergo oxidative decarboxylation to produce the final metabolites. Instead, the BCAAs and their corresponding alpha-ketoacids build up, leading to a metabolic crisis. MSUD: maple syrup urine disease, BCAAs: branched-chain amino acids, BCKDC: branched-chain ketoacid dehydrogenase complex

Our case highlights multiple important aspects in the individualized treatment of mild COVID-19 infection in a patient with MSUD, a relatively uncommon inborn error of metabolism that can be exacerbated by injury or infection. The metabolic pathway affected by MSUD is demonstrated in Figure 1.

The effects of COVID-19 infection in patients with MSUD have only been documented in two prior publications [6,7]. The first, an observational cohort study examining COVID-19 infection in patients with inborn errors of metabolism at a children’s hospital, reported a 26-year-old patient with MSUD who presented seven days after a positive COVID-19 test with encephalopathy and emesis after multiple days of poor appetite [6]. This patient’s leucine level peaked at 708 µmol/L, which was, for her, consistent with an acute metabolic crisis, and she was treated with a sick-day diet protocol and intravenous fluids, much like our patient. However, there was no mention of the utilization of remdesivir or other pharmacotherapies in this first case. The second publication, an observational survey-based study, examined the experiences of a Brazilian “rare disease community” with respect to COVID-19 infections [7]. This paper identified one patient with MSUD who was hospitalized with COVID-19 infection but who did not require intensive care or intubation. No data was reported regarding the specifics of his treatment while hospitalized. Based on these publications, little is known about how COVID-19 might uniquely affect patients with MSUD beyond the risk of metabolic crisis from infection.

Our case also brings up multiple important points regarding the unique pharmacologic treatment of our patient with MSUD during COVID-19 infection. As mentioned above, the patient’s immunocompromised state due to the medical management of her rheumatoid arthritis put her at an increased risk of developing a more severe COVID-19 course. For this reason, we decided to initiate remdesivir therapy with the goal of reducing the patient’s risk of progressing to more serious COVID-19 disease [4]. However, it quickly became evident that the patient’s worsening nausea and vomiting could be associated with remdesivir therapy. The risk of the patient progressing to a metabolic crisis from poor oral intake was greater than the risk of severe COVID-19 disease, especially given the patient’s lack of respiratory symptoms to that point. Remdesivir therapy was discontinued, and not only did the patient’s oral intake improve, but she also remained on room air and avoided progression to severe COVID-19 disease.

Another widely used pharmacotherapeutic agent in the treatment of COVID-19 in patients requiring supplemental oxygen is dexamethasone [8]. The primary reason for avoiding the use of dexamethasone, a glucocorticoid, in our patient was because glucocorticoids are known inducers of skeletal muscle protein breakdown and catabolism [9]. Glucocorticoid-induced catabolism had the potential to rapidly trigger a metabolic crisis in our patient with MSUD, so all members of the care team were informed of this contraindication. In the event of a respiratory decompensation requiring supplemental oxygen, we made a contingency plan to initiate baricitinib, an inhibitor of Janus (JAK) 1 and 2. Baricitinib has a mechanism of action distinct from glucocorticoids; it functions by inhibiting the cell signaling involved in the inflammatory pathways of various cytokines that tend to be elevated in severe COVID-19 infection [10] and therefore would pose little risk of triggering a metabolic crisis in our patient. In the case of respiratory decline due to COVID-19 pneumonia requiring mechanical ventilation in a patient with MSUD metabolic crisis, it would also be important to consider implementing the sick-day protein-restricted diet via specialized enteral formula to prevent neurologic consequences. No cases of MSUD metabolic crisis in the setting of COVID-19 infection requiring mechanical ventilation have been described in the literature. Fortunately, our patient suffered only a mild COVID-19 infection without respiratory symptoms nor the need for supplemental oxygen and additional pharmacotherapy. With close clinical monitoring and thoughtful input from our colleagues on the metabolic genetics team, we were able to prevent a metabolic crisis in our patient and discharge her home.

## Conclusions

The treatment of COVID-19 infection in a patient with MSUD requires a unique approach. Special care should be placed on maintaining adequate oral intake and closely monitoring amino acid levels to prevent progression to a metabolic crisis, especially in the setting of gastrointestinal distress. If the patient develops COVID-19 pneumonia with an oxygen requirement, via invasive or noninvasive means, glucocorticoids such as dexamethasone should be avoided, as they could trigger a metabolic crisis. Instead, nonsteroid options such as remdesivir and baricitinib should be considered if the patient is able to tolerate the side effects.
